# ﻿A taxonomic revision of *Garcinia* sections *Dicrananthera* and *Macrostigma* (Clusiaceae) in Thailand

**DOI:** 10.3897/phytokeys.248.130311

**Published:** 2024-11-07

**Authors:** Chatchai Ngernsaengsaruay, Pichet Chanton

**Affiliations:** 1 Department of Botany, Faculty of Science, Kasetsart University, Chatuchak, Bangkok 10900,Thailand; 2 Biodiversity Center, Kasetsart University (BDCKU), Chatuchak, Bangkok 10900, Thailand; 3 Suan Luang Rama IX Foundation, Nong Bon Subdistrict, Prawet District, Bangkok, 10250, Thailand

**Keywords:** Dioecy, *Discostigma*, edible fruits, Guttiferae, lectotypification, Malpighiales, taxonomy

## Abstract

*Garcinia* sections *Dicrananthera* and *Macrostigma* (Clusiaceae) is revised for Thailand. Three species are enumerated, i.e., one species, *G.thorelii* belongs to the sectionDicrananthera, and two species, *G.nuntasaenii* and *G.prainiana*, are in the sectionMacrostigma. Detailed morphological descriptions and illustrations are provided, together with notes on distribution, habitats and ecology, phenology, conservation assessments, etymology, vernacular names, uses, and specimens examined. An identification key to the species of sectionMacrostigma is presented. One name, *G.thorelii*, is lectotypified here. For *Garcinianuntasaenii* we recommend a conservation status of Endangered [EN B2ab(v)] and for the other two species (*G.prainiana* and *G.thorelii*) a conservation status of Least concern [LC]. A number of vegetative characters and features of reproductive organs, especially the flowers, distinguish the two sections and we enumerate these herein.

## ﻿Introduction

*Garcinia* L. is a group of evergreen trees, occasionally shrubs, which are usually dioecious but sometimes polygamo-dioecious. It also has obligately and facultatively agamospermous species. The color of latex secreted from cut boles, twigs, leaves, and fruits can be yellow, pale yellow, white, cream, or clear. The genus consists of c. 400 species (Gaudeul et al. 2024; POWO 2024), and is the largest genus in the Clusiaceae Lindl. (Guttiferae Juss.). It is a pantropically distributed genus and has centers of diversity located in Africa (Madagascar), Australasia, and Southeast Asia (Sweeney and Rogers 2008; Gaudeul et al. 2024). In Asia, *Garcinia* is most diverse in the Malesian region but also spreads north into southern China, west to India, and east to the Micronesian islands (Nazre et al. 2018).

The last monograph of the genus *Garcinia* was published by Engler (1893), who recognized 34 sections. Engler’s work was an elaboration of Pierre (1882, 1883), who established the first monograph of *Garcinia* and used mainly flower and inflorescence characters to classify the species into 37 sections. Vesque (1893) used floral morphology and leaf anatomy to classify the species into three subgenera and nine sections. A worldwide sectional treatment of *Garcinia* was presented by Jones (1980) in an unpublished Ph.D. thesis in which the genus was classified into 14 sections based largely on floral morphology, especially staminate flowers and pollen morphology. The latest is an updated infrageneric classification of the genus proposed by Gaudeul et al. (2024), who recovered nine major clades falling within two major lineages, and recognized 11 sections.

Gaudeul et al. (2024) resurrected Pierre’s (1882: t. 62) GarciniasectionDicrananthera for a morphologically coherent group of species that was designated the “*G.stipulata*” group in Sweeney et al. (2022). The section contains five species: *G.nujiangensis* C. Y. Wu & Y. H. Li, *G.paucinervis* Chun & F. C. How, *G.stipulata* T. Anderson, *G.thorelii* Pierre, and *G.yaatapsap* K. Armstr. & P. W. Sweeney. Jones (1980) placed *G.stipulata* and *G.thorelii* into sectionDiscostigma, creating a new subsection for them based on Pierre’s (1883: 8) sectional name *Dicrananthera* [GarciniasectionDiscostigmasubsectionDicrananthera (Pierre) S. W. Jones, *nom. inval.* following Art. 30.9 of the ICN (Turland et al. 2018)]. These species all share prominent stipuliform structures (rare in Clusiaceae, Stevens 2007), leaves with prominent, widely spaced, curved secondary veins and percurrent tertiaries, male flowers with numerous stamens united into an annular mass encircling and attached to the pistillode (in *G.paucinervis* and *G.nujiangensis* the stamens are described as being in four bundles [Chun and How 1956; Li 1981]), and ellipsoid fruits with a discoid stigma and one to two seeds (Sweeney et al. 2022).

*GarciniasectionMacrostigma* includes largely species that were included in Jones (1980) sections *Macrostigma*, *Mungotia*, and *Tripetalum*. This is a heterogenous group and it is difficult to find distinguishing features shared by all of the species in the section. Many species, especially those that were placed into sections *Macrostigma* and *Tripetalum*, often have stamen bundles adnate to the petals (Gaudeul et al. 2024). In the phylogeny, this clade includes three species that have been variously placed into other sections by other authors (Lauterbach 1922; Jones 1980): *G.hollrungii* Lauterb., *G.prainiana* King, and *G.warrenii* F. Muell. In addition to molecular data, these species have morphological characters that support their placement in sectionMacrostigma. This section was recently updated by Gaudeul et al. (2024), who recognized 29 species (e.g., *G.nuntasaenii* Ngerns. & Suddee, *G.phuongmaiensis* V. S. Dang, H. Toyama & D. L. A. Tuan). The section is distinguished by its staminate flowers lacking pistillode (usually, but rudimentary or well-developed pistillode present in some species); stamens united into central column (sometimes lobed with lobes equalling number of petals), or into completely separate antepetalous fascicles; androecium often adnate to the petals to varying degrees; two-thecous anthers; (three–) four to eight locular ovaries; unlobed and smooth or divided and papillose stigmas; fruits with smooth walls or faintly to deeply furrowed; and axillary or terminal inflorescences with one to many flowers (Gaudeul et al. 2024).

A taxonomic revision of *Garcinia* in Thailand has recently been undertaken by the first author as part of the *Flora of Thailand* project. More recently, Ngernsaengsaruay and Suddee (2016, 2022) described additional new species: *G.nuntasaenii* from North-Eastern and *G.santisukiana* Ngerns. & Suddee from Eastern Thailand, respectively. Ngernsaengsaruay (2022) recognized three species in GarciniasectionBrindonia (Thouars) Choisy in Thailand: *G.atroviridis* Griff. ex T. Anderson, *G.lanceifolia* Roxb., and *G.pedunculata* Roxb. ex Buch.-Ham. Ngernsaengsaruay et al. (2022a, 2023a) published additional new species records from Peninsular Thailand: *G.dumosa* King and *G.exigua* Nazre, respectively. Ngernsaengsaruay et al. (2022b) described *G.siripatanadilokii* Ngerns., Meeprom, Boonthasak, Chamch. & Sinbumr. as a new species from Peninsular Thailand. GarciniasectionXanthochymus (Roxb.) Pierre was revised for Thailand with four native species, i.e., *G.dulcis* (Roxb.) Kurz, *G.nervosa* (Miq.) Miq., *G.prainiana*, and *G.xanthochymus* Hook. f. ex T. Anderson (Ngernsaengsaruay et al. 2023b). GarciniasectionGarcinia was treated for Thailand with three species and one variety, i.e., two native species: *G.celebica* L. and *G.exigua* Nazre, and one cultivated species: G.mangostanaL.var.mangostana, including excluded and unplaced species, *G.anomala* Planch. & Triana (Ngernsaengsaruay et al. 2024a). Finally, Ngernsaengsaruay et al. (2024b) published an additional new species record from Peninsular Thailand, *G.minutiflora* Ridl.

From these publications, the genus has a total of c. 30 accepted species in Thailand. However, identifications mostly rely on the literature and the type specimens, and this is the case for *Garcinia* sections *Dicrananthera* and *Macrostigma*, which have never been revised for Thailand. Therefore, in this paper, we provide here an updated account for these two sections in Thailand in order to present a taxonomic treatment that includes lectotypification, detailed morphological descriptions, and illustrations, along with notes on distributions, habitats and ecology, phenology, conservation assessments, etymology, vernacular names, uses, and specimens examined. An identification key to the species of the sectionMacrostigma is presented.

## ﻿Materials and methods

The collected specimens were examined by consulting taxonomic literature (e.g., Pierre 1882, 1883; Vesque 1889, 1893; King 1890; Pitard 1910; Ridley 1922; Gagnepain 1943; Corner 1952; Whitmore 1973; Jones 1980; Ngernsaengsaruay and Suddee 2016; Ngernsaengsaruay et al. 2023b), and by comparing with herbarium specimens housed in the following herbaria: AAU, BK, BKF, BM, C, CMUB, K, P, PSU, QBG, and those included in the virtual herbarium databases of AAU (https://www.aubot.dk/search_form.php), CAL (https://ivh.bsi.gov.in/phanerogams), E (https://data.rbge.org.uk/search/herbarium/), K (including K-W) (http://www.kew.org/herbcat), L (https://bioportal.naturalis.nl/), P (https://science.mnhn.fr/institution/mnhn/collection/p/item/search/form), and US (https://collections.nmnh.si.edu/search/botany/). All herbaria acronyms follow Thiers (2024). All specimens cited have been seen by the authors unless stated otherwise. The taxonomic history of the species was compiled using the taxonomic literature and online databases (IPNI 2024; POWO 2024). The morphological characters, distribution, habitats and ecology, phenology, and uses were described from historic and newly collected herbarium specimens and the author’s observations during field work. The vernacular names were compiled from the specimens examined and the literature (e.g., Pooma and Suddee 2014; Ngernsaengsaruay and Suddee 2016; Ngernsaengsaruay et al. 2023b). Thailand floristic regions follow *Flora of Thailand* Vol 4(3.3) (The Forest Herbarium, Department of National Parks, Wildlife and Plant Conservation, 2023). The assessment of conservation status was performed following the IUCN Red List Categories and Criteria (IUCN Standards and Petitions Committee 2022) to yield a preliminary assessment of the conservation category in combination with GeoCAT analysis (Bachman et al. 2011) and field information. The calculation of Extent of Occurrence (EOO) and Area of Occupancy (AOO) are based on GeoCAT (https://www.kew.org/science/our-science/projects/geocat-geospatial-conservation-assessment-tool).

## ﻿Results and discussion

### ﻿Taxonomic treatment

**Sectional classification sensu Gaudeul et al. (2024)**

#### ﻿ Garcinia section Dicrananthera

Taxon classificationPlantaeMalpighialesClusiaceae

Pierre, Fl. Forest. Cochinch. 1(5): 8. 1883; M. Gaudeul et al., PhytoKeys 239: 95. 2024.

848E1832-E437-57E8-8AA9-1369A4D3641D

##### Type.

*Garciniathorelii* Pierre, Fl. Forest. Cochinch. 1(4): t. 62. 1882.

GarciniasectionDicrananthera is distinguished by the presence of a pair of interpetiolar stipuliform structures (rare in Clusiaceae); leaves with prominent, widely spaced secondary veins and scalariform tertiary veins; axillary or terminal cymose inflorescences with three to many flowers [i.e., *G.yaatapsap* (3–5-flowered), *G.paucinervis* (4–10-flowered), *G.stipulata* (4–6-flowered), *G.nujiangensis* (6–10-flowered), and *G.thorelii* (20–40-flowered thyrse)]; flowers with 4 sepals and 4 petals; male flowers with numerous stamens united into an annular mass encircling and attached to the pistillode (i.e., *G.stipulata* and *G.yaatapsap*) or united into 4 bundles surrounding the pistillode (i.e., *G.nujiangensis*, *G.paucinervis*, and *G.thorelii*), 2-thecous anthers; 1–2-locular ovaries; unlobed and smooth stigmas; and fruits with a smooth surface and unlobed. Distinguishing morphological characters reported here for this section were taken from Pierre 1882, Pitard 1910, Chun and How 1956, Jones 1980, Li 1981, Li et al. 2007, Stevens 2007, Gaudeul et al. 2024, and from our observations.

A section of five species worldwide (Gaudeul et al. 2024); one species in Thailand.

#### ﻿ Garcinia thorelii

Taxon classificationPlantaeMalpighialesClusiaceae

Pierre, Fl. Forest. Cochinch. 1(4): t. 62. 1882; Vesque, Epharmosis 2: 16. t. 146. fig. 77. 1889 et in A. DC. & C. DC., Monogr. Phan. 8: 367. 1893; Engl. in Engl. & Prantl, Die Naturlichen Pflanzenfamilien 3(6): 236. 1893; Pit. in Lecomte et al., Fl. Indo-Chine 1(4): 301. 1910; Craib, Fl. Siam. 1(1): 118. 1925; Gagnep. in Gagnep., Fl. Indo-Chine Suppl.: 260. 1943; S. W. Jones, Morphology and Major Taxonomy of Garcinia (Guttiferae), Ph.D. Thesis (unpublished): 375. fig. 7/13. 1980.

1FDFE9AA-A70D-59CB-B98D-D22DF551CCBE

Figs 1 , 2 , 3

##### Type.

Laos • Paklai, fl. & fr., Expedition Mekong 1866–1868, *C. Thorel 3365* (lectotype designated here P [P04701082!]; isolectotypes [P04701076!, P04701080!, P04701081!, P04701083!], K [K000677688!].

**Figure 1.**
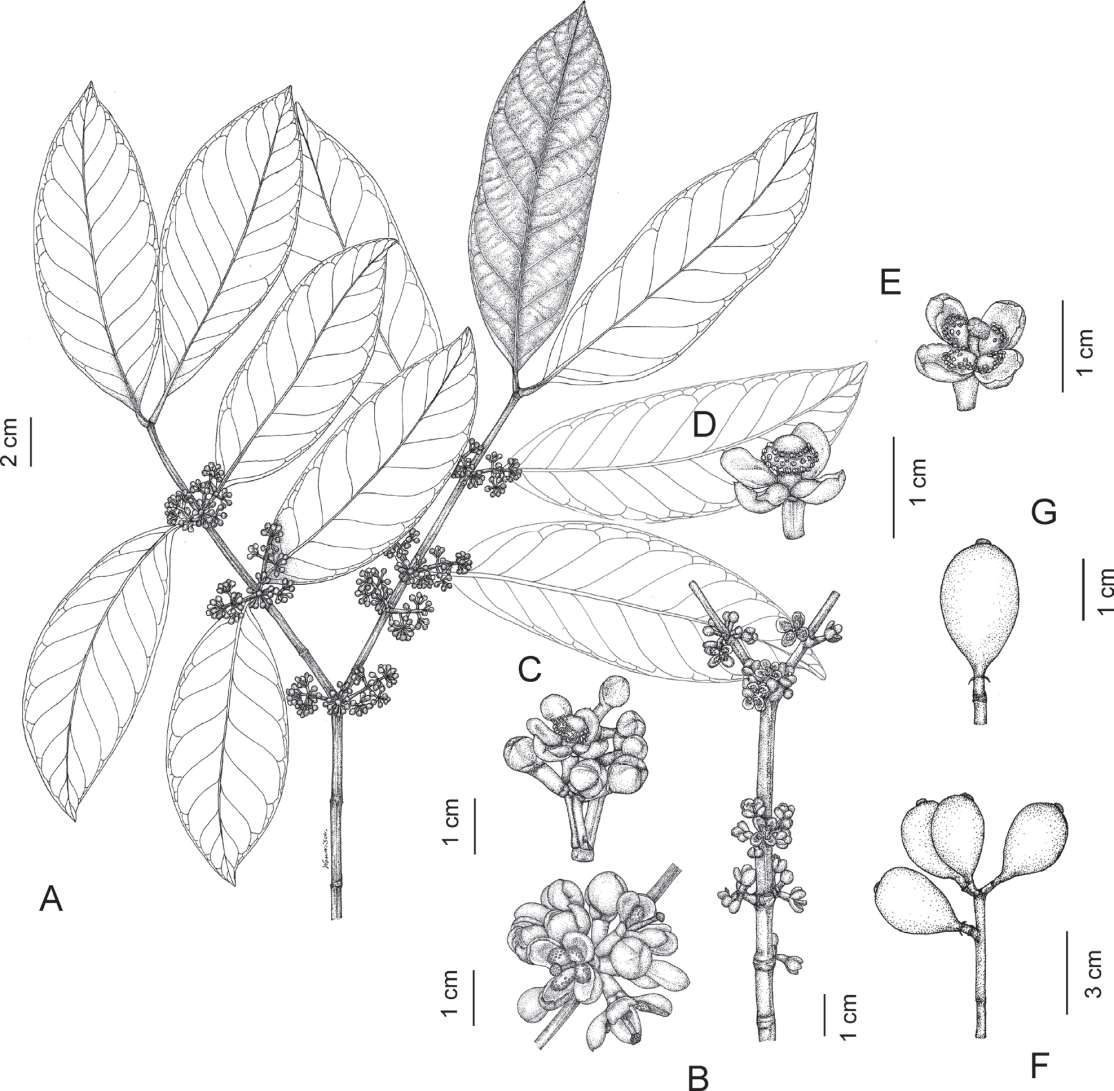
*Garciniathorelii***A** branchlets, leaves, and male inflorescences **B** branchlets and male inflorescences with male flower buds and fully open male flowers **C** male inflorescences with male flower buds and early stage of open male flower **D** early stage of open male flower **E** fully open male flower showing 4 petals and numerous stamens united into 4 bundles surrounding a pistillode **F** infructescence **G** fruit. Photo: Drawn by Wanwisa Bhuchaisri.

##### Description.

***Habit*** evergreen tree, 8–20 m tall, 50–180 cm GBH; latex yellow, sticky; branches decussate, horizontal or nearly horizontal; branchlets green, 4-angular, glabrous. ***Bark*** reddish brown or brown, cracked or shallowly fissured; inner bark pale brown. ***Terminal bud*** concealed between the bases of the uppermost pair of petioles. ***Stipuliform structures*** 2, interpetiolar, caducous, triangular, 1.2–2.7 × 1–2.8 mm, apex acute. ***Leaves*** decussate; lamina elliptic, narrowly elliptic or oblanceolate-obovate, 7.5–18 × 3–7.5 cm, apex acuminate or acute, base cuneate or obtuse, margin repand, coriaceous, dark green above, paler below, glabrous on both surfaces, midrib shallowly grooved or flattened above, raised below, secondary veins 5–10 each side, curving towards the margin and connected in distinct loops and united into an intramarginal vein, flattened above, slightly raised below, intersecondary veins usually absent, tertiary veins scalariform, veinlets reticulate, visible below, interrupted long wavy lines (glandular wavy lines, also called exudate containing canals) present, of differing lengths, running across the secondary veins to the apex, faint; petiole green, 0.6–1.5 cm long, grooved above, slightly transversely rugose, glabrous, with a basal appendage clasping the branchlet. ***Inflorescences*** axillary or at leafless nodes (in axils of fallen leaves), a short thyrse of many flowers, 2–2.5 cm long; bracts caducous, triangular, 0.6–2 mm long, apex acute; peduncle 5–8 mm long, 4-angular; rachis 0.8–2 cm long, 4-angular. ***Flowers*** unisexual, plants dioecious, 4-merous, fully open flowers 0.5–1.5 cm in diam.; bracteoles caducous, triangular, 0.8–1.5 mm long, apex acute; pedicels 2–4 mm long; sepals 4 and petals 4, decussate, glabrous; sepals pale green, not concave, triangular, 1–2.5 × 1–2.5 mm, subequal, apex obtuse; petals pale yellow or creamish white, concave, suborbicular, obovate or broadly elliptic, 3–5.5 × 2–4.5 mm, subequal, apex rounded, margin irregularly dentate. ***Male flowers***: stamens numerous united into 4 bundles surrounding a pistillode, bundles 1.3–2.8 × 0.6–2 mm; filaments very short; anthers small; pistillode fungiform (mushroom-shaped), 2–3 mm long; rudimentary ovary slender, cylindrical, 0.5–1.5 mm long; sterile stigma pale yellow, sessile, convex, unlobed, 0.8–1.5 mm in diam., papillate. ***Female flowers***: staminodes absent; pistil fungiform, 2.5–3 mm long; ovary ellipsoid, c. 1.5 mm long, glabrous, 1–2-locular; stigma convex, unlobed, 1.5–2 mm in diam., smooth. ***Fruits*** berries, ellipsoid or broadly ellipsoid, 1.5–2.5 × 1–2 cm, green, smooth, glabrous, with persistent sepals; persistent stigma convex, unlobed, 2.5–4 mm in diam., smooth; fruiting stalks 2–5 mm long, glabrous. ***Seeds*** 1, ellipsoid, 1.5–2 × 1–1.2 cm, with a thin fleshy pulp.

**Figure 2.**
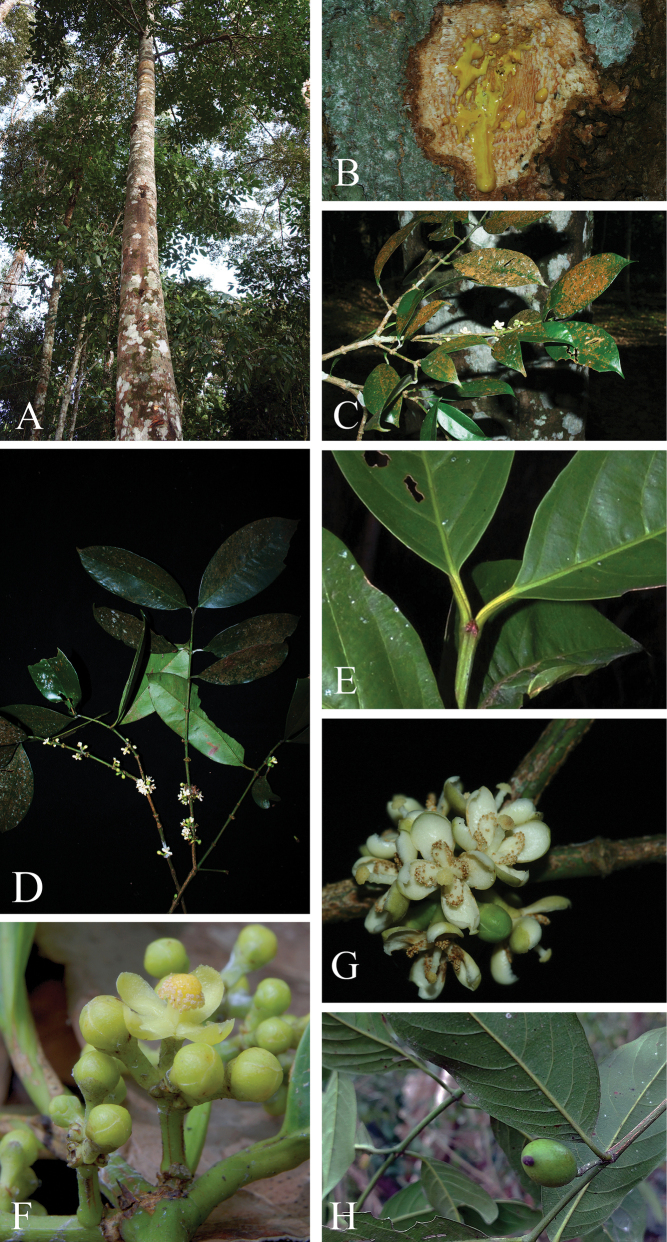
*Garciniathorelii***A** habit and stem **B** slashed bark with yellow latex **C, D** branchlets, leaves, and male inflorescences with male flower buds and open male flowers **E** interpetiolar stipuliform structure **F** branchlets and male inflorescences with male flower buds and early stage of open male flower **G** branchlets and male inflorescences with male flower bud and fully open male flowers showing 4 petals and numerous stamens united into 4 bundles surrounding a pistillode **H** branchlets, leaves, and fruit. Photos: Chatchai Ngernsaengsaruay (**A–G**), Yotsawate Sirichamorn (**H**).

**Figure 3.**
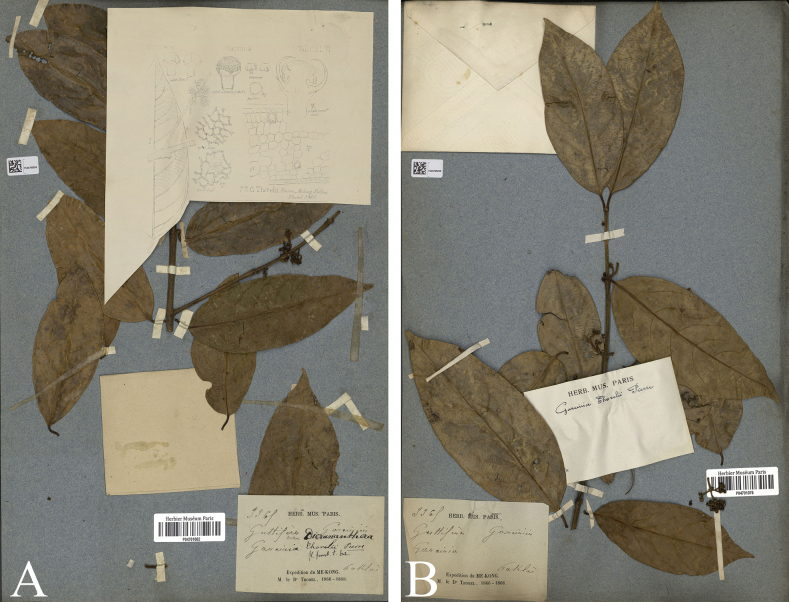
Types of *Garciniathorelii*, *C. Thorel 3365* from Paklai, Laos, Expedition Mekong 1866–1868 **A** lectotype designated here P [P04701082!] **B** isolectotype P [P04701076!]. Photos: Muséum National d’Histoire Naturelle, Paris, France, http://coldb.mnhn.fr/catalognumber/mnhn/p/p04701082, http://coldb.mnhn.fr/catalognumber/mnhn/p/p04701076.

##### Distribution.

**Figure 4.**
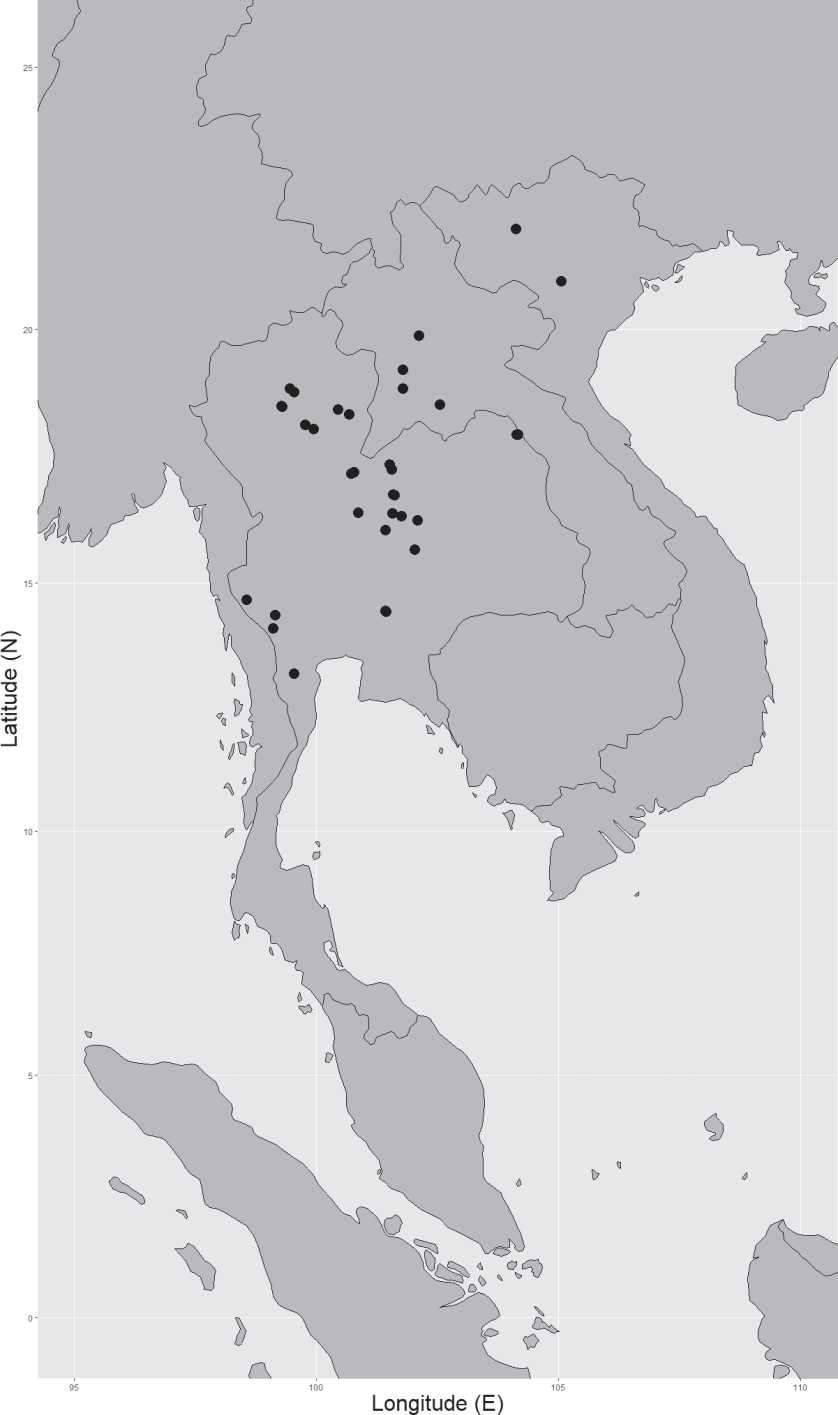
Distribution of *Garciniathorelii*, known from Vietnam, Laos, and Thailand. In Thailand, this species is known to be naturally distributed in the northern, the north-eastern, the eastern, and the south-western regions.

Vietnam, Laos, and Thailand (Fig. 4).

##### Distribution in Thailand.

**Northern**: Nan, Lamphun, Lampang, Phrae, Phitsanulok; **North-Eastern**: Phetchabun, Loei, Bueng Kan, Nakhon Phanom; **Eastern**: Chaiyaphum, Nakhon Ratchasima; **South-Western**: Kanchanaburi, Phetchaburi (Fig. 4).

##### Habitat and ecology.

It is found in dry evergreen forests, lower montane rain forests, mixed deciduous forests, often along streams, sometimes on limestone hills, 50–1,300 m amsl.

##### Phenology.

Flowering and fruiting more than once, nearly throughout the year.

##### Conservation status.

*Garciniathorelii* is distributed from Vietnam to Laos and Thailand. It is known from many localities and has a large EOO of 338,548.61 km^2^ and a small AOO of 128 km^2^. In Thailand, this species is known to be naturally distributed in four floristic regions and 13 provinces and has an EOO of 145,632.54 km^2^ and an AOO of 96 km^2^. Because of its number of localities and because it does not face any threat of extinction we therefore suggest the conservation status Least Concern (LC).

##### Etymology.

The specific epithet of *Garciniathorelii* honors Clovis Thorel (1833–1911), a French physician, botanist, and plant collector (Stafleu and Cowan 1986), who collected the type specimen of this species.

##### Vernacular names.

Kok (ก๊อก) (Phitsanulok, from the specimen *A. F. G. Kerr 5816*); Khrak khamin (ครากขมิ้น), **Ma da khi non** (มะดะขี้หนอน) (Northern); Mai khwak lueang (ไม้ขวากเหลือง) [Phrae, from the specimen *Unknown s.n.* (BKF40333)]; Khi non (ขี้หนอน) (Laos, from the specimen *J. E. Vidal 5952*).

##### Uses.

The wood is used for house construction [from the specimen *Unknown s.n.* (BKF40333)].

##### Lectotypifications.

*Garciniathorelii* was named by Pierre (1882: t. 62), who cited the specimen *Thorel 3365* collected from Laos, Paklai. He did not choose a holotype nor did he mention the name of the herbarium where the specimen was housed. However, we located five sheets of *C. Thorel 3365* at P [P04701076, P04701080, P04701081, P04701082, P04701083] and one sheet at K [K000677688], and following Art. 9.6 of the ICN (Turland et al. 2018), these are syntypes. The P [P04701082] specimen is the best preserved and is selected here as the lectotype, following Art. 9.3 and 9.12 of the ICN (Turland et al. 2018).

##### Notes.

According to Jones 1980, the shape and size of leaves of *Garciniathorelii* are narrowly elliptic, ovate or obovate and 18–20 × 6–9 cm. However, from our observations, we found the shape and size of leaves of this species can be elliptic, narrowly elliptic or oblanceolate-obovate and sometimes smaller, 7.5–18 × 3–7.5 cm.

According to Jones 1980, male flowers of this species have numerous stamens united into an annular mass around and attached to a pistillode in a narrow ring halfway up. However, from our examinations, the stamens are united into 4 bundles surrounding a pistillode. In the early stage of open flowers, the stamen bundles are attached to a pistillode (not spreading), then spreading in the fully open flowers.

According to Jones 1980, the shape and size of fruits of this species is narrowly ellipsoid and c. 1.5 × 0.6 cm. From our examinations, we found the shape and size of fruits of this species are ellipsoid or broadly ellipsoid and larger, 1.5–2.5 × 1–2 cm.

##### Additional specimens examined.

**Thailand. Northern** • Nan [Nam Haeng, young fr., 14 Jul 1926, *Winit 1751* (BKF, K [K003964565])] • Lamphun [along the trail to Tat Moei Waterfall, Doi Khun Tan National Park, Mae Tha District, fl., 29 Jun 1993 (as *Garciniahanburyi*), *J. F. Maxwell 93-728* (BKF, CMUB, L [L2403744, L2403745]) • Doi Khun Tan National Park, fl., 29 Apr 1994 (as *G.hanburyi*), *J. F. Maxwell 94-562* (BKF, CMUB)] • Lampang [Mae Saloi, fl., 29 Oct 1925, *Winit 1492* (BKF, K [K003964566]) • Chae Son National Park, Wang Nuea District, ♂ fl., 2 Jun 1996 (as *G.hanburyi*), *J. F. Maxwell 96-792* (BKF, CMUB) • ibid., sterile, 3 Jun 2014 (as *G.hanburyi*), *T. Riythiwigrom 1* (CMUB)] • Phrae [Locality unspecified, fl., 1912, *Luang Vanpruk 279* (BKF, K [K003964569]) • Locality unspecified, fl., s.d., *Luang Vanpruk 514* (E [E00839797], K [K003964567]) • Huai Ta, Mae Song, fl., 28 May 1912, *Luang Vanpruk 303* (BKF, K [K003964568]) • Long District, fl., 12 Mar 1930, *Winit 1909* (BKF, K [K003964570]) • Thung Laeng Subdistrict, Long District, fr., 4 Feb 1963 (*Garcinia* sp.), *Unknown s.n.* (BKF40333) • Huai Rong Waterfall, fr., 19 Jun 1996 (as *Garcinia* sp.), *R. Pooma & P. Puudjaa 1400-1* (BKF) • Phitsanulok [Nakhon Thai District, fl., 11 Apr 1922, *A. F. G. Kerr 5816* (BM, E [E00839798], K [K003964572], P [P04701077]) • Huai Sai, fl., 18 Jun 1967 (as *Garcinia* sp.), *S. Phusomsaeng 241* (BKF, K [K003964575], P [P05062032]) • Political and Military School, Phu Hin Rong Kla National Park, Nakhon Thai District, ♂ fl., 30 Sep 2007, *C. Ngernsaengsaruay G56-30092007* (BKF, spirit material)] • Province unspecified [Pa Hia, near Pang Pue, fl., 28 Mar 1914, *A. F. G. Kerr 3174* (BM, E [E00160901], K [K003964571])]; **North-Eastern** • Phetchabun [Khao Paya Paw, fr., 4 Mar 1931, *A. F. G. Kerr 20342* (BKF, BM, C, E [E00160902], K) • Nam Nao National Park, fr., 8 Apr 1976 (*Garcinia* sp.), *Bunnak 3108* (BKF) • Wang Pong District, fr., 1 Feb 2001, *T. Wongprasert 012-3* (BKF, L [L3811042]) • Huai Phrom Laeng, Nam Nao National Park, fl., 18 Jan 2003 (as *Garcinia* sp.), *P. Chantaranothai et al. 512003* (AAU) • along stream, Nam Nao National Park, fl., 28 May 2013 (as *Garcinia* sp.), *C. Maknoi 5589* (QBG)] • Loei [Phu Luang Wildlife Sanctuary, sterile, 17 May 1998 (as *Garcinia* sp.), *K. Chayamarit et al. 1480* (BKF) • Huai Baeng Forest Protection Station, Phu Luang Wildlife Sanctuary, Wang Saphung District, fr., 22 Jun 2003, *T. Wongprasert 036-47* (BKF)] • Bueng Kan [Bueng Khong Long, Kinnari Waterfall, Phu Langka National Park, fr., 20 May 2014, *S. Sirimongkol et al. 596* (BKF, K [K003964580], L [L4367330])] • Nakhon Phanom [Tat Kham Waterfall, Phu Langka National Park, fr., 30 Oct 1998, *T. Wongprasert s.n.* (BKF120854)]; **Eastern** • Chaiyaphum [Nam Phrom, fr., 10 Dec 1971 (as *Garcinia* sp.), *C. F. van Beusekom et al. 4085* (BKF, C, K [K003964578], P [P05062035]) • Ban Nam Phrom, fr., 24 May 1974 (as *Garcinia* sp.), *R. Geesink et al. 6910* (BKF, K [K003964574], L [L2409515]) • Phu Khiao, young fr., 3 Aug 1972 (*Garcinia* sp.), *K. Larsen et al. 31312* (BK, K [K003964576], L [L2409542]) • Phu Khiao Wildlife Sanctuary, fr., 8 Nov 1984 (as *Garcinia* sp.), *G. Murata T-50251* (BKF) • ibid., fr., 3 May 1997 (as *Garcinia* sp.), *R. Pooma 1539* (BKF, CMUB) • Tabo–Huai Yai Wildlife Sanctuary, fr., 22 Aug 2019 (as *Garcinia* sp.), *N. Boonruang 0350* (QBG)] • Nakhon Ratchasima [Koa Lem, fl. & fr., 12 Jan 1925, *A. F. G. Kerr 9978* (BM, E [E00839799], K [K003964573], P [P04701078]), *9979* (BM, P [P04701079]) • Khao Yai National Park, fl. & fr., 9 Apr 1974, *T. Smitinand 12001* (BKF)]; **South-Western** • Kanchanaburi [Erawan Waterfall, fl., 25 Jan 1962 (as *Garcinia* sp.), *K. Larsen & T. Smitinand 9271* (BKF, C, K [K003964577]) • Sai Yok, fr., 26 Nov 1971 (as *Garcinia* sp.), *C. F. van Beusekom et al. 3987* (BK, C, K [K003964579], P [P05062045]) • Vajiralongkorn Dam, Tha Khanun Subdistrict, Thong Pha Phum District, fl., 11 Jan 1985 (as *Garcinia* sp.), *H. Koyama T-49019* (BKF)] • Phetchaburi [Mae Kradang La Waterfall, Kaeng Krachan National Park, Nong Ya Plong District, female fl. & fr., Feb 2024, *Y. Sirichamorn* personal observation with photos].

**Vietnam** • Tonkin [prov. de Lauyson, fr., 19 Dec 1913 (as *Garcinia* sp.), *Unknown 29663* (P [P04788166]) • Hoa-Binh, fr., 1 Nov 1929 (as *G.bonii*), *M. Brillet unreadable number* (P [P04899809, P04899810])].

**Laos** • Luang Phrabang [Locality unspecified, fl., 20 Mar 1932 (as *Garcinia* sp.), *Poilane 20452* (P [P04899801])] • Sayaboury [Mekong River, Na Konken Village, ♂ fl., 28 Apr 2012, *J. F. Maxwell 12-154* (CMUB, L [L4345648])] • Vientiane [Reservoir Nam Ngum, fr., 19 Oct 1974 (*Garcinia* sp.), *J. E. Vidal 5952* (P [P05061722, P05061726])].

#### ﻿ Garcinia section Macrostigma

Taxon classificationPlantaeMalpighialesClusiaceae

Pierre, Fl. Forest. Cochinch. 1(6): 63. 1883; M. Gaudeul et al., PhytoKeys 239: 94. 2024.

16B5AEF4-00BE-5EBE-96DA-9CED6629CA00

##### Type.

*Garcinialatissima* Miq., Ann. Mus. Bot. Lugduno-Batavi 1: 209. 1864.

GarciniasectionMacrostigma is characterized by its axillary or terminal cymose inflorescences with two to many flowers (in a simple cyme or in fascicles of several simple cymes), or a solitary flower (in female flowers); 4(–5)-merous flowers [4-merous, e.g., *G.nuntasaenii*; 5-merous, e.g., *G.phuongmaiensis*, *G.prainiana*]; male flowers with numerous stamens united into a central column, sometimes lobed with lobes equaling number of petals [a single weakly 4-lobed bundle, e.g., *G.nuntasaenii*], or into completely separate antepetalous bundles [united into 5 bundles, e.g., *G.phuongmaiensis*, *G.prainiana*]; androecium often adnate to the petals to varying degrees; 2-thecous anthers; pistillode absent in male flowers, but rudimentary or well-developed pistillode present in some species; (–3)4–8-locular ovaries; unlobed or lobed and smooth or papillate stigmas; and fruits with a smooth surface, unlobed or faintly, shallowly or deeply lobed. Distinguishing morphological characters of this section based on Gaudeul et al. 2024, which includes additional information.

A section of 29 species worldwide (Gaudeul et al. 2024); two species in Thailand (i.e., *G.nuntasaenii* and *G.prainiana*).

### ﻿A key to the species of GarciniasectionMacrostigma in Thailand

A related species from Vietnam is included in the key.

**Table:** 

1	Shrub; leaves smaller (4–17 × 2.5–7.5 cm); flowers smaller (0.8–1 cm in diam.); petals pale yellow, creamish white or white; fruits lobed, turning red or bright red when ripe; distributed in Central Vietnam, Central Laos, and North-Eastern Thailand	**2**
–	Tree; leaves larger (12.5–27.5 × 5.5–11.5 cm); flowers larger (2.5–4 cm in diam.); petals variable in color: pale yellow, yellowish pink, yellowish red, pinkish red, pink or red; fruits unlobed, turning bright yellow, orangish yellow and bright orange when ripe; distributed in Peninsular Malaysia and Peninsular Thailand	***Garciniaprainiana* (2.)**
2	Flowers 4-merous; petals pale yellow or creamish white; fruits 4–6-lobed; distributed in Central Laos and North-Eastern Thailand	***Garcinianuntasaenii* (1.)**
–	Flowers (4–)5-merous; petals white; fruits 3–4-lobed; distributed in Central Vietnam	***Garciniaphuongmaiensis***

#### ﻿1. Garcinia nuntasaenii

Taxon classificationPlantaeMalpighialesClusiaceae

Ngerns. & Suddee, Thai Forest Bull., Bot. 44(2): 134. figs 1, 2, 3. 2016; Tagane et al., Edinburgh J. Bot. 75(1): 110. fig. 2G. 2018.

D5FC3CF0-9433-530A-86FA-D95ABDDD8B9A

Fig. 5

##### Type.

Thailand • Bueng Kan Province, Bung Khla District, Phu Wua Wildlife Sanctuary, fr., 13 Dec 2008, *N. Nuntasaen 10* (holotype BKF!; isotype BKF!).

**Figure 5.**
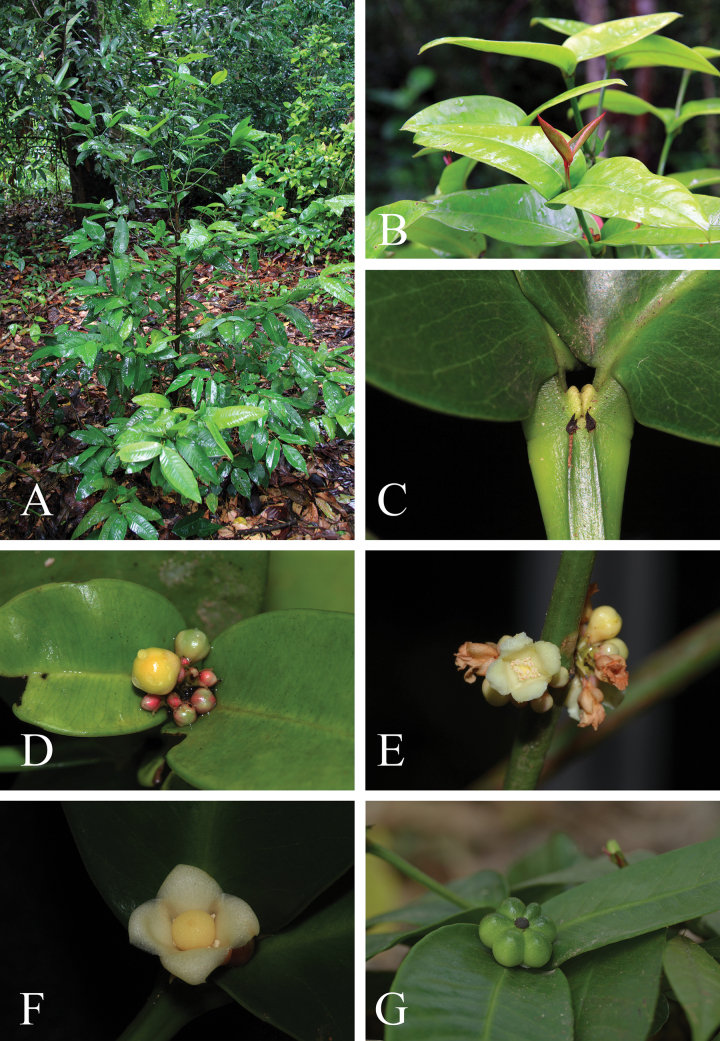
*Garcinianuntasaenii***A** habit and habitat **B** branchlets and young leaves **C** terminal bud concealed between the bases of the uppermost pair of petioles **D** terminal inflorescences with flower buds **E** branchlets and male inflorescences in axils of fallen leaves with male flower buds and open male flowers **F** female flower (top view) **G** branchlets, leaves, and fruit. Photos: Chatchai Ngernsaengsaruay (**A–D, F**), Manop Poopath (**E, G**).

##### Description.

***Habit*** shrub, 1–2 m tall; latex white, turning pale yellow, sticky; branches decussate, horizontal or nearly horizontal; branchlets green, 4-angular, glabrous. ***Bark*** green when young, turning dark brown when mature, smooth, usually lenticellate; inner bark pale yellow. ***Terminal bud*** concealed between the bases of the uppermost pair of petioles. ***Leaves*** decussate; lamina lanceolate-ovate, ovate or elliptic, 6–17 × 3–7.5 cm, apex acuminate or acute and rigid, base subcordate, margin thick, entire and slightly undulate (repand), coriaceous, slightly bullate or bullate, apical part of leaves conduplicate, twisted and recurved, glossy dark green above, paler below, glabrous on both surfaces, midrib shallowly grooved above, raised as a prominent ridge below, secondary veins 12–20 each side, curving towards the margin and connected in distinct loops and united into an intramarginal vein, flattened above, slightly raised below, conspicuous on both surfaces, intersecondary veins conspicuous, veinlets reticulate, visible on both surfaces, interrupted long wavy lines present, of differing lengths, nearly parallel to the midrib, running across the secondary veins to the apex, visible below; petiole green, 0.2–1 cm long, not grooved, transversely rugose, glabrous, with a basal appendage clasping the branchlet; young leaves red. ***Inflorescences*** terminal or at leafless nodes (in axils of fallen leaves), cymose. ***Flowers*** unisexual, plants dioecious, 4-merous, fully open flowers 0.8–1 cm in diam.; bracteoles narrowly triangular or triangular, 2–10 × 1.5–2.5 mm, apex acute or acuminate, somewhat thick; pedicels short; sepals 4 and petals 4, decussate, pale yellow or creamish white, somewhat thick; sepals obovate or elliptic, 3–6 × 2.5–3.5 mm, concave, apex rounded; petals broadly obovate, 5.5–8.5 × 3.5–7 mm, apex rounded, apical part recurved. ***Flower buds***: sepals pink or pink-pale yellow. ***Male flowers*** in fascicles of several simple cymes; stamens numerous, united into a single weakly 4-lobed bundle; filaments very short; anthers 0.3–0.5 mm long; pistillode usually absent. ***Female flowers*** usually in a cluster of 3 flowers (a simple cyme); staminodes numerous, united into a single weakly 4-lobed bundle, surrounding the ovary; pistil fungiform; ovary depressed globose, 1.5–2 × 2–3 mm, shallowly 4–6-lobed, 4–6-locular; stigma convex, hemispherical, 1.5–2 × 2.5–3.5 mm, papillate. ***Fruits*** berries, depressed globose, 0.5–0.7 × 1–2 cm, 4–6-lobed, green with white dots, turning red when ripe, smooth, glabrous, glossy, with persistent sepals; persistent stigma circular, flat, radiately lobed or unlobed; fruiting stalk 2–4 mm. long. ***Seeds*** 4–6, with fleshy pulp. The description of this species is based on Ngernsaengsaruay and Suddee 2016, which includes additional information.

##### Distribution.

Central Laos and North-Eastern Thailand.

##### Distribution in Thailand.

**North-Eastern**: Bueng Kan, Nakhon Phanom.

##### Habitat and ecology.

It is found in dry evergreen forests, 150–220 m amsl.

##### Phenology.

Flowering and fruiting more than once; flowering in December to July; fruiting in December to April.

##### Conservation status.

*Garcinianuntasaenii* is known only from four localities in Bueng Kan and Nakhon Phanom Provinces, North-Eastern Thailand and Nam Kading National Protected Area, Laos. All occurrences are in small populations. It has a small EOO of 796.87 km^2^ and a relatively small AOO of 16 km^2^. Because of its number of localities, and because the roots of this species are used for medicinal purpose by nearby villagers in Thailand, the population is suspected to be declining. We therefore suggest the conservation status Endangered [EN B2ab(v)].

##### Etymology.

The specific epithet of *Garcinianuntasaenii* honours Mr Narong Nuntasaen, a staff member and a plant collector of BKF, who collected the type specimen.

##### Vernacular names.

**Chang nga ek** (ช้างงาเอก) (Bueng Kan, Nakhon Phanom).

##### Uses.

The fruits are edible (from the specimen *M. Norsaengsri & N. Tathana 8630*). The roots are locally used for medicinal purposes (Ngernsaengsaruay personal observation).

##### Note.

*Garcinianuntasaenii* is similar to *G.phuongmaiensis* in its habit (shrubs); sticky white latex, turning pale yellow when exposed to the air; 4-angular branchlets, especially when young; coriaceous, bullate, shiny dark green, subcordate leaves with a short petiole; flower size, c. 1 cm in diam.; and the color of fruits, turning red when ripe, but differs in having 4-merous flowers [vs (4–)5-merous flowers]; pale yellow or creamish white petals (vs white petals); 4–6-lobed fruits (vs 3–4-lobed fruits); and is also distributed in Central Laos and North-Eastern Thailand (vs distributed in Central Vietnam). The morphological characteristics and distribution of *G.phuongmaiensis* were taken from Tuan et al. (2023).

##### Additional specimens examined.

**Thailand. North-Eastern** • Bueng Kan [Phu Wua Wildlife Sanctuary, Bung Khla District, fl., 8 Nov 1996 (*Garcinia* sp.), *C. Niyomdham 4910* (BKF) • ibid., fl., 27 Aug. 2001 (*Garcinia* sp.), *R. Pooma et al. 2791* (BKF) • ibid., ♂ fl., 1 Jan 2008 (spirit specimen), *N. Nuntasaen 11* (BKF) • ibid., fr., 27 Dec 2011 (*Garcinia* sp.), *M. Norsaengsri & N. Tathana 8630* (BKF, QBG) • ibid., 12 Feb 2015, *M. Poopath 981-1, 981-2* (BKF) • ibid., ♀ fl., 24 Jul 2015, *C. Ngernsaengsaruay & N. Meeprom 754* (BKF) • The Upper Northeast Wild Plants Conservation Center, Bung Khla District, ♂ fl., 24 Jul 2015, *C. Ngernsaengsaruay & N. Meeprom 750, 751* (BKF) • ibid., ♀ fl., 24 Jul 2015, *C. Ngernsaengsaruay & N. Meeprom 752, 753* (BKF)] • Nakhon Phanom [Ban Phaeng District, Phu Langka National Park (Narong Nuntasaen own observation)].

**Laos** • Bolikhamxay [Nam Kading National Protected Area, ♂ fl., *L8* • ibid., fl. & fr., *L431* • ibid., *L1034* (Tagane et al. 2018).

#### ﻿2. Garcinia prainiana

Taxon classificationPlantaeMalpighialesClusiaceae

King, J. Asiat. Soc. Bengal, Pt. 2, Nat. Hist. 59(2): 171. 1890; Vesque in A. DC. & C. DC., Monogr. Phan. 8: 329. 1893; Ridl., Fl. Malay Penins. 1: 180. 1922; Corner, Wayside Trees Mal. 1: 320. fig. 112. ed. 2. 1952; Corner & Watan., Ill. Guide Trop. Pl.: t. 193. 1969; Whitmore in Whitmore, Tree Fl. Malaya 2: 220. 1973; I. M. Turner, Gard. Bull. Singapore 47(1): 263. 1995; Ngernsaengsaruay et al., PeerJ, DOI 10.7717/peerj.16572: 28. figs 13, 14, 15. 2023.

6D5026F1-9176-5A6D-B8F6-C721079CB3DC

Fig. 6

##### Type.

Peninsular Malaysia • Perak, Kuala Dipang (originally ‘‘Kwala Dipang’’ on the label; originally published ‘‘Kwala Dynong’’), fl., February 1885, *B. Scortechini 1796* (lectotype designated by Ngernsaengsaruay et al. 2023b: 28, CAL [CAL0000005844, photo seen]; isolectotypes K [K000677678!], P [P04701324, photo seen]).

**Figure 6.**
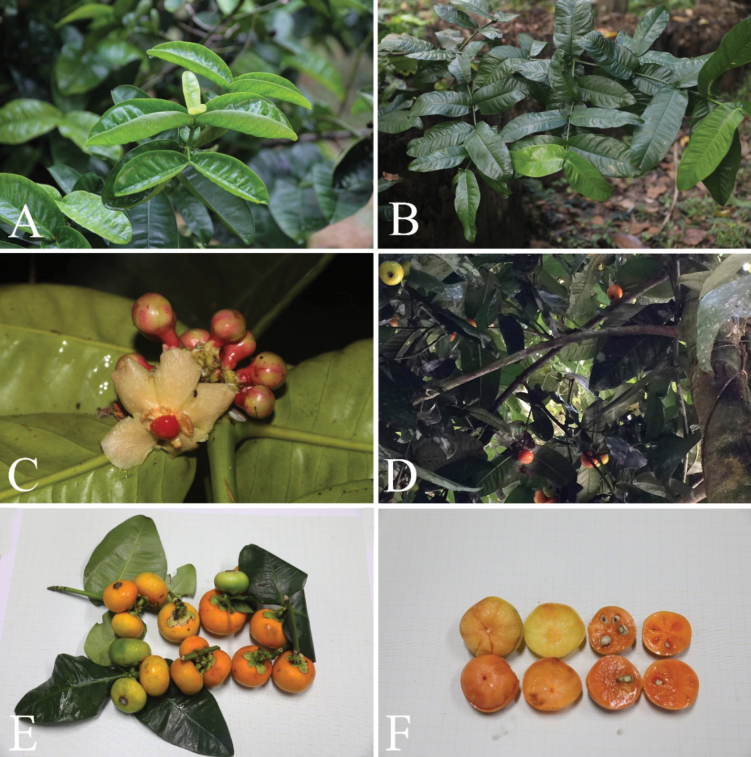
*Garciniaprainiana***A, B** branchlets, young leaves, and mature leaves **C** inflorescences with male flower buds and open male flower **D** branches and branchlets with mature and ripe fruits **E** branchlets, leaves, and mature and ripe fruits **F** ripe fruits (pericarp removed and in transverse section showing seeds with fleshy pulp). Photos: Chatchai Ngernsaengsaruay (**A–C, E, F**), G Rawit Sichaikhan (**D**).

##### Description.

***Habit*** evergreen trees, 3–12 m tall, 15–75 cm GBH; latex white, sticky; branches decussate, horizontal or nearly horizontal; branchlets 4-ridged, glabrous. ***Bark*** pale brown, grayish brown or blackish brown, smooth or slightly rough; inner bark pale yellow. ***Terminal bud*** concealed between the bases of the uppermost pair of petioles. ***Leaves*** decussate; lamina elliptic, oblong or elliptic-oblong, sometimes narrowly oblong, 12.5–27.5 × 5.5–11.5 cm, apex acute or obtuse, base subcordate, often subamplexicaul, margin repand and slightly revolute, coriaceous, bullate or slightly bullate, shiny dark green above, paler below, glabrous on both surfaces, midrib flattened above, raised as a prominent ridge below, secondary veins 9–20 each side, curving towards the margin and connected in distinct loops and united into an intramarginal vein, flattened above, raised below, conspicuous on both surfaces, intersecondary veins conspicuous, veinlets reticulate, visible on both surfaces, interrupted long wavy lines present, of differing lengths, nearly parallel to the midrib, running across the secondary veins to the apex, visible below; petiole green, 1.5–6 mm long, 2–5 mm in diam., not grooved, transversely rugose, glabrous, with a basal appendage clasping the branchlet; young leaves shiny pale green. ***Inflorescences*** terminal, sometimes on short, leafless lateral branchlets, cymose, usually in dense fascicles of several to many flowers. ***Flowers*** unisexual, plants dioecious, 5-merous, fully open flowers with spreading petals; bracteolate; sepals 5 and petals 5, quincuncial, coriaceous, glabrous. ***Male flowers*** 2.5–3.5 cm in diam.; bracteoles pale green, triangular 2.3–4.5 × 1.8–3.7 mm, apex acute, conduplicate with a central keel; pedicel pinkish green, reddish green or greenish red, 3–6 mm long, 2.5–4 mm in diam., widened at the apical part, terete, glabrous; sepals pinkish green, reddish green or greenish red, concave, broadly ovate or suborbicular 4.8–8 × 5–7.8 mm, unequal, apex rounded; petals variable in color: pale yellow, yellowish pink, yellowish red, pinkish red, pink or red, broadly obovate or obovate, 0.8–1.4 × 0.6–1.1 cm, subequal, sometimes unequal, apex rounded; stamens numerous, united into 5 bundles surrounding a pistillode, antepetalous, 1.7–4.2 mm long, each bundle 1.2–4 mm wide, pale yellow, pink or red; filaments fused throughout their entire length; anthers yellow, 0.3–0.6 mm long; pistillode fungiform, 5.5–7.5 mm long; sterile stigma pale yellow, pink or red, sessile, convex, indistinctly lobed, 5–6 mm in diam., papillate. ***Female flowers*** 2.5–4 cm in diam.; bracteoles and pedicel same as in male flowers; sepals and petals same as or slightly larger than in male flowers; staminodes absent; pistil fungiform, 6–8.5 mm long; ovary pale green, depressed globose 4–6 × 4.5–6.5 mm, unlobed, glabrous, 5–8-locular; stigma pale yellow, pink or red, sessile, convex, weakly 5–8-lobed or indistinctly lobed, 5–7 mm in diam., papillate. ***Fruits*** berries, depressed globose or depressed subglobose, sometimes globose, 2–3.5 × 2–5.3 cm, sometimes oblique, asymmetrical, unlobed, slightly concave or flattened at the apex, green, turning greenish yellow, bright yellow, orangish yellow and bright orange when ripe, smooth, glabrous, glossy, then exocarp becoming dark brownish black and slightly sinuously wrinkled when dry, pericarp 3.5–8 mm thick, exocarp thin; persistent stigma dark brown or blackish brown, circular, button-like, 0.6–1.1 cm in diam., slightly concave or flattened, weakly 5–8-lobed or indistinctly lobed, papillate; persistent sepals pale green, turning yellowish green and orangish green, larger than in flowering material; fruiting stalk green, thick, 0.4–1.5 cm long, 3–6 mm in diam., ***Seeds*** 1–6, often aborted, brown, broadly ellipsoid, ellipsoid or subglobose, 0.9–1.6 × 0.7–1.4 cm, with pale orange fleshy pulp. The description of this species was taken from Ngernsaengsaruay et al. 2023b.

##### Distribution.

Known only from Peninsular Thailand and Peninsular Malaysia. It is widely distributed in Peninsular Malaysia (Perlis, Kedah, Penang, Perak, Kelantan, Terengganu, Pahang, Selangor, Negeri Sembilan, Malacca, Johor) (Corner 1952; Whitmore 1973; Turner 1995; Azuan and Salma 2018). It can be found mainly in Pahang, Perak, and Negeri Sembilan (Syazwani 2020).

##### Distribution in Thailand.

**Peninsular**: Yala (Than To), Narathiwat (Waeng, Su-ngai Kolok).

##### Habitat and ecology.

It is found in tropical lowland evergreen rain forests, occasionally along streams, 30–200 m amsl. It is also cultivated in villages and botanical gardens.

In Peninsular Malaysia, it occurs in lowland and hill forests, on hillsides and ridges up to elevations of 1,000 m amsl. It is also cultivated in villages (Whitmore 1973; Syazwani 2020; from the specimen *T. C. Whitmore Kep. FRI4018*).

##### Phenology.

Flowering and fruiting more than once; flowering nearly throughout the year, usually in February to May; fruiting April to June and September to December.

##### Conservation status.

LC (Ngernsaengsaruay et al. 2023b).

##### Etymology.

The specific epithet of *Garciniaprainiana* refers to Sir David Prain (1857–1944), a British botanist, a herbarium curator of the Royal Botanic Garden, Calcutta (1887–1898), and a director of the Royal Botanic Gardens, Kew (1905–1922) (Stafleu and Cowan 1983).

##### Vernacular names.

**Chupu** (จูปู) (Malay-Narathiwat); Cerapu, Chekau, Chepu, Cherapu, Cherpu, Cherupu, Chupak, Chupu, Kechupu, Kecupu, Menchepu, Menchupu (Malay); Button mangosteen (English).

##### Uses.

*Garciniaprainiana* is locally cultivated for its fruits in peninsular Thailand. The fruits (pericarp and fleshy pulp surrounding the seeds) are edible and have a sour or sweet-sour taste. It is also grown in some botanical gardens as an ornamental plant to provide botanical education.

In Peninsular Malaysia, it is commonly cultivated in village gardens. The ripe fruits are edible and are sometimes used fresh in beverages (Allen 1965; Burkill et al. 1966). The pulp of fruits has high antioxidant content of about 91.9% and vitamin C content of about 27.3 mg per 100 g fresh weight (Azuan and Salma 2018). In a traditional Malay recipe, the raw fruits are described as being cooked with dried fish (Zawiah and Othaman 2012). The wood is used for house building (Allen 1965; Burkill et al. 1966). It is an excellent ornamental plant for use in landscape gardens in parks (National Parks Flora and Fauna Web 2023).

##### Notes.

According to Ngernsaengsaruay et al. (2023b), the male flowers of *Garciniaprainiana* were reported to have a small ring-shaped disk surrounding the base of the pistillode. However, in this study, we re-examined the flowers, and a small ring-shaped disk is absent.

*Garciniaprainiana* is also similar to *G.phuongmaiensis* in having coriaceous, bullate, shiny dark green, subcordate, subamplexicaul leaves with a short petiole; 5-merous flowers; and numerous stamens, united into 5 antepetalous bundles surrounding a pistillode, but differs in relatively larger habit as a 3–12 m tall tree (vs smaller habit, shrubs, 1–3 m tall); larger leaves, 12.5–27.5 × 5.5–11.5 cm (vs smaller leaves, 4–11 × 2.5–5 cm); larger flowers, 2.5–4 cm in diam. (vs smaller flowers, c. 1 cm in diam.); variable in color of petals: pale yellow, yellowish pink, yellowish red, pinkish red, pink or red (vs white petals); staminodes absent (vs present); unlobed fruits, turning bright yellow, orangish yellow and bright orange when ripe (vs shallowly 3–4-lobed fruits, turning bright red when ripe); seeds with pale orange fleshy pulp (vs seeds with white fleshy pulp); and is distributed in Peninsular Malaysia and Peninsular Thailand (vs Central Vietnam). The morphological characteristics and distribution of *G.phuongmaiensis* were taken from Tuan et al. (2023).

Vesque (1893) placed *Garciniaprainiana* with species of G.sectionXanthochymus (subgenus Xanthochymus), and this placement was followed by Jones (1980). However, the flowers of *G.prainiana* have a pistillode and lack receptacular disks and antepetalous appendages, unlike those found in other G.sectionXanthochymus species (e.g., *G.dulcis*, *G.subelliptica* Merr.) or other species in “lineage A”. These flowers, with staminal phalanges adnate to the petals, and *G.prainiana*’s branching, adaxial, exudate-containing canal pattern agree with the molecular data and support its placement within a subclade (clade 9) of “lineage B” with which it shares many features (Sweeney 2008). More recently, Gaudeul et al. (2024) reported that in addition to molecular data, *G.prainiana* has morphology that supports its placement into G.sectionMacrostigma.

##### Additional specimens examined.

**Thailand. Central** • Nakhon Nayok [Phrueksaphan Thepparat Botanicical Garden, Chulachomklao Royal Military Academy, cultivated, 31 May 2019, *C. Ngernsaengsaruay & W. Boonthasak G30-31052019* (BKF); **Peninsular** • Trang [Khao Chong Botanical Garden, Chong Subdistrict, Na Yong District, cultivated, 16 Feb 2022, *C. Ngernsaengsaruay et al. G32-16022022* (BKF)] • Yala [Chulabhorn Phatthana 7 Project, Than To District, near waterfall, 27 Nov 2019, *C. Ngernsaengsaruay & G. Sichaikhan G31-27112019* (BKF)] • Narathiwat [Hala-BalaWildlife Sanctuary, Ban Bala, Lo Chut Subdistrict, Waeng District, ♂ fl., 13 May 2005 (as *Garcinia* sp.), *M. Poopath 274* (BKF) • Hala-Bala Wildlife Sanctuary, Waeng District, fr., 22 Sep 2005, *C. Niyomdham & P. Puudjaa 7593* (BKF) • Su-ngai Kolok District, fr., 20 Apr 2002, *U. Upho 556* (QBG) • Su-ngai Kolok District, fl., cultivated, 20 May 2003, *U. Upho 550* (BKF)].

**Peninsular Malaysia** • Perak [Kwala Dipang, ♂ fl., Dec 1896, *C. Curtis 3273* (K [K000677679]) • Kg Kepayang near Ipoh, fr., 30 Oct 1971, *Syed Abu Bakar Kep. FRI20440* (L [L2417220])] • Pahang [Su-ngai Bertam at Kuala Mensum, fl., 2 Jun 1971, *T. C. Whitmore Kep. FRI20091* (L [L2417222]) • Path leading to Kuala Mensum from Boh Tea, Cameron Highlands, fr. 24 Sep 1971, *H. S. Loh Kep. FRI19187* (L [L2417221]) • Cameron Highlands Road, fr., 18 Jan 1982, *K. M. Kochummen Kep. FRI29377* (L [L2417225]) • Kelantan [0.5 mile east of Gua Musang, fr., 14 Jul 1967, *T. C. Whitmore Kep. FRI4018* (L [L2417226]) • Su-ngai Lebir, below Kuala Relai at Jentah, fl., 24 Apr 1976, *B. C. Stone & M. Sidek 12426* (BKF, L [L2417224], US [US02961246]) • Su-ngai Long off Su-ngai Pergau, Jeli, fr., 26 Sep 1986, *A. Latiff et al. ALM1856* (L [L3806490], PSU) • Ketam, Cicar Tinggi, Kampung Bata, Pasir Mas, ♀ fl. & fr., 1 Aug 1992, *A. Noorsiha et al. Kep. FRI39214* (L [L3878683]) • Pasir Putih, fl., 23 Oct 1992, *H. Husmady et al. Kep. FRI39551* (L [L3806959]) • near Brooke Camp, Gua Musang, fl., 2 Jun 1994, *H. Husmady et al. Kep. FRI41841* (L [L2417223])].

## Supplementary Material

XML Treatment for Garcinia section Dicrananthera

XML Treatment for Garcinia thorelii

XML Treatment for Garcinia section Macrostigma

XML Treatment for Garcinia nuntasaenii

XML Treatment for Garcinia prainiana

## ﻿Additional information

### Author ORCIDs

Chatchai Ngernsaengsaruay https://orcid.org/0000-0002-7131-976X

Pichet Chanton https://orcid.org/0009-0001-7325-6109

### Data availability

All of the data that support the findings of this study are available in the main text.
